# The Fellowship of the Royal College of Surgeons

**Published:** 1914-09-05

**Authors:** 


### THE FELLOWSHIP OF THE ROYAL COLLEGE OF SURGEONS.

The Fellowship of the Royal College of Surgeons of England is justly esteemed as the premier qualification that the student can obtain in surgery, and is almost an essential to anyone who aspires to the higher surgical posts in England. The student should decide early in his career whether he wishes to obtain the F.R.C.S. For this purpose it is necessary to pass two examinations, a primary and a final.

The Primary examination takes place twice every year, in May and in November, and is held in London. The subjects are anatomy and physiology, and candidates must have passed the second Conjoint examination or some other equivalent examination which exempts them from it. They must also (except in the case of members of the college) have spent three winter sessions or eighteen months in dissections and attendance at physiology classes at a recognised school. The fee for the examination is £5 5s. The examination is partly oral and partly written. Four papers are set, two in physiology and two in anatomy, four questions, of which only two must be answered, being given with an allowance of two hours for each paper. The oral examinations are two, lasting twenty minutes each, in the two subjects. The examination has the reputation of being unusually stiff, but its difficulty should not frighten the student or practitioner of reasonable ability who is willing to pay special attention to the two subjects. A good, sound knowledge of anatomy and physiology is required, and if the candidate has been diligent at his practical work in the dissecting room and laboratory, and has supplemented the knowledge there gained by reading, he stands a good chance of success.

The examination for the Final Fellowship takes place in May and November of each year, and consists of a written paper, *viva voce*, clinical examinations, and operative surgery. The fee for the examination is £12 12s., and candidates who are members of the college are admissible when they have been six years in practice or in the study of medicine. Other candidates must show that they are graduates in medicine of recognised Universities of four years' standing, and have attended the surgical practice of a recognised hospital for one year after graduation. Special classes for the Final Fellowship examination are held at most of the London hospitals; and practitioners who intend to enter for it, and have lost touch to any extent with hospital surgical practice, would do well to attend a full course of instruction at one of the medical schools before competing.

